# A mobile MRI field study of the biochemical cartilage reaction of the knee joint during a 4,486 km transcontinental multistage ultra-marathon using T2* mapping

**DOI:** 10.1038/s41598-020-64994-2

**Published:** 2020-05-18

**Authors:** Uwe Schütz, Martin Ehrhardt, Sabine Göd, Christian Billich, Meinrad Beer, Siegfried Trattnig

**Affiliations:** 1 Department of Diagnostic and Interventional Radiology, University Hospital of Ulm, Albert-Einstein-Allee 23, D-89081 Ulm, Germany; 2 MR Centre of Excellence- High Field MR Centre, Department of Biomedical Imaging and Image-Guided Therapy, Medical University of Vienna, BT32, Lazarettgasse 14, 1090 Vienna, Austria

**Keywords:** Magnetic resonance imaging, Outcomes research

## Abstract

Nearly nothing is known about the consequences of ultra-long-distance running on knee cartilage. In this mobile MRI field study, we analysed the biochemical effects of a 4,486 km transcontinental multistage ultra-marathon on femorotibial joint (FTJ) cartilage. Serial MRI data were acquired from 22 subjects (20 male, 18 finisher) using a 1.5 T MR scanner mounted on a 38-ton trailer, travelling with the participants of the TransEurope FootRace (TEFR) day by day over 64 stages. The statistical analyses focused on intrachondral T2* behaviour during the course of the TEFR as the main outcome variable of interest. T2* mapping (sagittal FLASH T2* weighted gradient echo) is a validated and highly accurate method for quantitative compositional cartilage analysis of specific weightbearing areas of the FTJ. T2* mapping is sensitive to changes in the equilibrium of free intrachondral water, which depends on the content and orientation of collagen and the proteoglycan content in the extracellular cartilage matrix. Within the first 1,100 km, a significant running load-induced T2* increase occurred in all joint regions: 44.0% femoral-lateral, 42.9% tibial-lateral, 34.9% femoral-medial, and 25.1% tibial-medial. Osteochondral lesions showed no relevant changes or new occurrence during the TEFR. The reasons for stopping the race were not associated with knee problems. As no further T2* elevation was found in the second half of the TEFR but a decreasing T2* trend (recovery) was observed after the 3,500 km run, we assume that no further softening of the cartilage occurs with ongoing running burden over ultra-long distances extending 4,500 km. Instead, we assume the ability of the FTJ cartilage matrix to reorganize and adapt to the load.

## Introduction

The question of whether endurance running poses an increased risk of knee osteoarthritis (OA) remains controversial^1–3^. A number of authors state that prolonged running is not associated with an increased prevalence of cartilage degeneration^4–10^, while others indicate a risk for OA^1,11–14^. Chakravarty *et al*.^15^ found no acceleration of radiographic knee OA in healthy older runners (50–71 years). Due to the non-pathological, but highly repetitive strain on the legs, excessive running stress can be regarded as mild chronic trauma to the joints of the lower extremities. Prolonged running burden over time is thought to alter the collagen network and lubricating proteoglycans, slowly wear out the articular cartilage, and cause microfractures in the subchondral bone^4,16^. However, this hypothesis has not been proven, and clinical and radiographic cohort studies of regular long-distance running subjects, up to marathons, have shown no significance regarding knee OA incidence and progression in hip and knee joints without knee injury, poor muscle tone, obesity, or proprioceptive deficit^9,17–24^.

The physiology of ultra-endurance exercise is very important because it is believed that the ability to walk and run long distances has played an important role in human evolution^25^. The German Ultramarathon Association defines a multistage ultra-marathon (MSUM) as a marathon with more than 4 stages, each with a distance of more than 50 km. However, although the amount of data pertaining to long-distance running has increased in recent decades, little is known about the consequences of ultra-long-distance running, such as MSUM, on knee joint cartilage^26^.

Over the last 2 decades, magnetic resonance imaging (MRI) has become a validated and reliable *in vivo* method for quantitative and qualitative assessment of human joint cartilage^27–29^. Quantification of changes in the composition of hyaline cartilage during short (5 km) and long-distance runs (marathon) can be carried out noninvasively by mapping the T1rho- and T2-relaxation times^30–40^. As these values are elevated in patients with OA, these mapping methods have played increasingly important role in detecting the onset of cartilage degeneration^40–43^. T1rho mapping shows a high negative correlation with intrachondral proteoglycan concentration and appears to be more sensitive to concentration changes^44,45^. T2 mapping, however, is sensitive to changes in the equilibrium of free water, which depend on the orientation and content of collagen and the content of proteoglycans in the extracellular cartilage matrix^31,41,43,46–50^.

According to the anisotropic properties of the collagen fibres in articular cartilage, the T2 relaxation changes with different spatial orientations in the magnetic field of the MR scanner^51^. In the femorotibial joint (FTJ), T2 value inequalities and variabilities between corresponding cartilage regions are correlated with the limited water mobility within an anisotropic rigid matrix^52^. They mostly depend on the different degrees of stiffness and creep rates^53^ needed in relation to degrees of biomechanical stress and loading (cartilage compression)^54^, resulting in regionally different glycosaminoglycan (GAG) content and collagen fibril network orientation^55^. It is widely accepted that load-induced intrachondral T2 increases result from a loss of structural anisotropy of type II collagen matrix (spatial orientation of collagen fibrils) and from an increase in free cartilage water^41,47,56,57^.

As a correlate of the disturbance of the cartilage structure and composition, Mosher *et al*. showed that running induced an initially significant decrease in the superficial chondral T2 values in the FTJ^37^. Subburaj *et al*.^42^ also described a significant decrease in T1rho- and T2-relaxation times after only 30 min of running, which indicates that an increased cartilage running load leads to a temporary dehydration of the chondral tissue. Luke *et al*. evaluated an increase in chondral T1rho and T2 values in the first days after a single marathon compared with a control group^58^, and the highest signal changes were found in the medial FTJ in the marathon runners.

With shorter measurement times and possible 3D recordings, the T2* technique^59–61^, which is also related to the collagen network and the water content, has been shown to be a practical alternative to the time-consuming T2 technique with multiple echo times^59,62^. In addition, T2* imaging offers an inherently higher signal-to-noise ratio (SNR) and greater robustness^63,64^ than the other methods (T1rho/T2 mapping, delayed gadolinium-enhanced MRI of cartilage (dGEMRIC)^61^). As contrast media is not required, T2* is considered to be the best and only viable option for mobile MRI field studies, such as the TransEurope-FootRace (TEFR) project^65^.

The TEFR project, which evaluated participants in the transcontinental MSUM from the southernmost (Bari, Italy) to the northernmost border of Europe (North Cape), is the first study to show the feasibility of recording mobile MRI from ultra-marathon runners over 4,486 km through 6 countries and a period of 64-days without any day of rest; the mean stage distance was 70.1 km or 1.7 marathons (min 44 km, max 95.1 km)^65^. The prospective approach applied in this long-term MRI field study led to unique data accumulation, revealing in detail the influence of an ultra-endurance burden on different tissues^66–71^.

The main purpose of this article was to characterize the effect of MSUM running on the intrachondral free water distribution and collagen fibre content and organization using serial MRI (T2* response) recordings over several weeks. The results may provide evidence that ultra-running alters the extracellular cartilage matrix behaviour of the FTJ towards cumulative structural chondral damage with an increased risk of OA. Furthermore, we evaluated whether any detectable running-induced T2* reactions show regional differences in FTJ cartilage.

## Methods

### Study population

After approval by the local ethics committee (University Hospital of Ulm, No.: 270/08-UBB/se) and obtaining written informed consent, 22 TEFR participants (demographics see Table 1) were included in the mobile MRI study of the knee joints. All methods were performed in accordance with the relevant guidelines and regulations. The inclusion criterion was official acceptance as a participant at the TEFR by the organizers^65^, including age> 17 years, the presence of a medical certificate indicating physical health, and the successful completion of at least one MSUM. No subject had to be excluded due to contraindications against native MRI^72^. The subjects had no relevant knee injuries (meniscus tears, cruciate ligament ruptures, etc.) or prior surgical interventions of the knee joints, which could change the intrinsic cartilage conditions and thus normal chondral T2 signalling^65^. The baseline data (before the start of the marathon) of the 18 of the 22 subjects (81.8%) who remained in the race until the last measurement interval (MI) were as follows: mean age of 51.0 years (SD 10.4, range 26–65), 17 males (94.4%), body mass of 70.4 kg (SD 7.5, range 58.9–82.2), body mass index (BMI) of 23.0 kg/m² (SD 1.8, range 20.3–26.4), and body height of 175 cm (SD 6, range 163–184). Reasons for premature termination of the TEFR were overuse reactions of the soft tissues of the lower extremities (“shin splints”, myofascial inflammation) in 2 male participants, stress fracture of the left ventral pelvic ring in one female participant, and compliance problems by one male participant.

**Table 1:** Subjects demographics of TEFR-project at baseline (t0).

	TEFR-No.	m/f	age (yrs)	BMI (kg)	leg preference	finisher/non-finisher (finished stages)	total run time (hrs)
**1**	01	m	43.8	25.4	right	F	415.9
**2**	09	f	45.6	22.4	right	F	692.7
**3**	10	m	53.1	22.4	right	F	626.6
**4**	13	m	52.4	23.7	right	F	515.3
**5**	16	f	46.4	21.2	left	NF (49)	(419.2)
**6**	19	m	30.8	24.6	right	F	560.7
**7**	20	m	57.2	20.2	right	F	615.2
**8**	22	m	50.7	21.8	right	NF (28)	(208.6)
**9**	26	m	56.6	24.3	right	F	651.5
**10**	32	m	26.2	20.6	right	F	407.0
**11**	33	m	65.4	26.4	left	F	645.3
**12**	34	m	63.2	22.2	right	F	669.3
**13**	40	m	53.1	22.4	right	F	484.9
**14**	50	m	65.1	21.3	left	F	650.0
**15**	51	m	46.1	22.2	right	F	548.5
**16**	55	m	53.0	21.1	right	F	545.3
**17**	57	m	43.5	20.8	right	F (left study at stage 42)	(642.2)
**18**	59	m	49.9	25.6	right	F	506.7
**19**	60	m	58.5	21.6	right	F	629.0
**20**	63	m	49.4	24.6	right	F	517.3
**21**	64	m	48.9	22.2	right	F	566.1
**22**	68	m	46.4	24.1	left	NF (10)	(38.2)

### Data acquisition

MRI data were acquired with a mobile 1.5 T MR scanner (Magnetom Avanto^TM^ mobile MRI 02.05, software version: Syngo^TM^ MR B15, Siemens Ltd., Germany) mounted on an MRI trailer (Model Mob. MRI 02.05, SMIT Mobile Equipment B.V., Farnham, UK), which was pulled by a truck tractor (38 tonnes in total) and travelled with the runners throughout entire TEFR from stage to stage, day by day^65^. MRI scanning was planned at baseline (t0; within the 4 days prior to the start of the TEFR), at 3 MIs during the TEFR (t1 planned after 1,200 km, t2 after 2,400 km, and t3 after 3,600 km of the run; between 2:00 pm and 9:00 pm after the daily stage), and 7 months after the TEFR (tx). However, this plan was not able to be implemented consistently during the field study. Influencing factors such as daily changing weather conditions, local conditions, and the individual changing needs and sensitivities of the subjects due to the immense physical and mental stress within the framework of such an MSUM required daily modification and rescheduling of the scanning plan^65^. The actual measurement intervals implemented during the TEFR project were as follows: T1 was between stages 14–20 after a mean distance run of 1,103 km (SD 106 km, range 926–1,325 km), T2 was between stages 39–45 after a mean distance run of 2,779 km (SD 218 km, range 2,313–3,082 km), and T3 was between stages 51–57 after a mean distance run of 3,673 km (SD 234 km, range 3,516–3,973 km). To minimize variation in joint positioning, a table-fixed, 8-channel knee coil was used. In the finisher group, 17 subjects (77.3%) underwent MRI at all MIs (t0-t3), and 12 of them underwent MRI at tx. T2* mapping was performed with a sagittal fast low-angle shot T2* weighted gradient-echo sequence with a 60° flip angle, echo times of 4.2/12.2/19.9/27.7/35.4 ms, a repetition time of 1,120 ms, a slice thickness of 3.0 mm, a field of view of 289 cm², a pixel size of 0.11 mm², and an in-plane resolution of 0.332 iso. T2* relaxation times were calculated from online reconstructed T2* maps by using a pixelwise, mono-exponential non-negative least squares fit analysis (syngo^TM^ MapIt; Siemens Ltd.)^50,73^. Sagittal turbo inversion recovery measurements and coronal fat saturated proton density weighted sequences were also performed.

### Image evaluation

**Figure 1:**
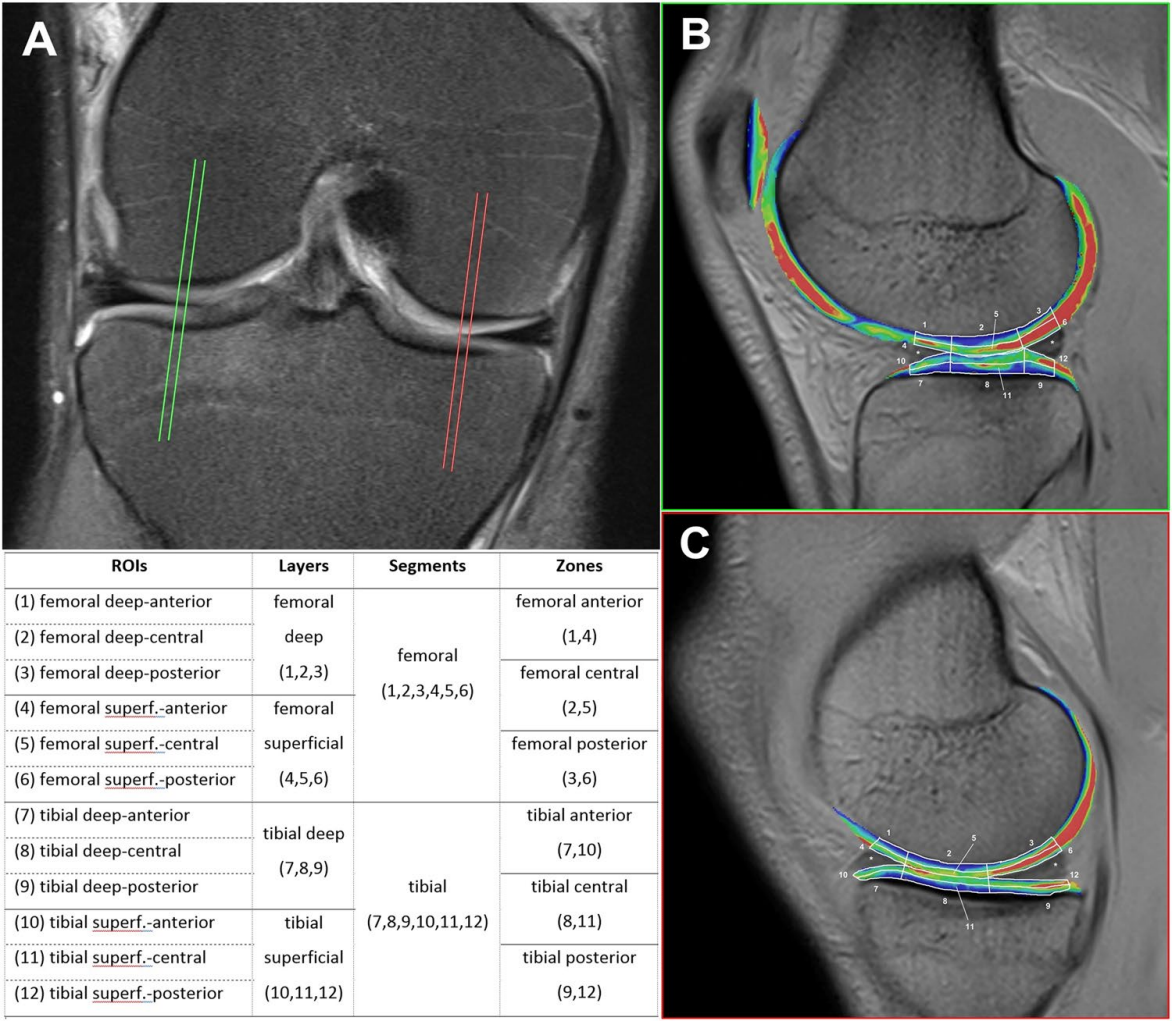
MR-image postprocessing for quantification of cartilage T2: (**A**) 4 sagittal slices (2 median slices in lateral and medial FTJ); **B** + **C:** fused colored T2* maps of 2 median sagittal FLASH T2* GRE (**C**) lateral FTJ, (**D**) medial FTJ).

For measurements of cartilage T2* profiles, 4 sagittal slices through the FTJ were determined, and the 2 most median slices in the medial and lateral femorotibial compartment were used. To determine if there were regional differences in the T2* response of cartilage to ultra-running burden, a subset quantitative analysis was performed in which cartilage regions of interest (ROIs) were separately generated from the medial and lateral knee compartments^50^. These ROIs were manually drawn on the 4 sagittal slices such that the whole femoral and tibial cartilage area was covered between the meniscal bases (see Fig. 1). These areas were separated into a superficial and a deep layer and in an anterior (cartilage covered by the anterior meniscal horn), central (uncovered cartilage between the anterior and posterior meniscal horn) and posterior zone (cartilage covered by the posterior meniscal horn)^74^. In total, the representative cartilage T2* map was separated into 12 ROIs on each slice (see Fig. 1). Care was taken to avoid the subchondral bone or joint fluid and to set the ROIs in the exact same positions at every examination. The ROIs were drawn twice by 2 independent investigators with a time gap of 6 months. They were supervised by 2 radiologists with 30 to 15 years of experience in musculoskeletal imaging. As a strong significant correlation (*p* < 0.01) was found between the 2 series (r > 0.8) for all cartilage areas and most of the ROIs (see Supplementary Table S1), averaged data of the mean T2* values of the 2 series were taken for further analysis. To determine the mean T2* for each cartilage area (layers, zones, or segments), the mean T2* values of the specific ROIs were pooled and calculated with regard to the ROI sizes (see Supplementary Table S2). Sufficient intra- and interobserver precision and reliability results of the T2* measurement method used herein have already been presented^29,34,41,50,62,70,71^. The precision (reproducibility) of the drawn ROI sizes in the course of the TEFR was calculated with 3.2 CV% in mean (SD 0.24) between the MIs (t0-t3) and no significant changes in the drawn ROI areas were detected for all investigated ROIs.

### Statistical analysis

For data documentation, analysis and graphical presentation, Microsoft^TM^ Excel^TM^ (Office-Business 2016), SPSS^TM^ (IBM^TM^-Statistics, release 25.0, 2017) and SigmaPlot^TM^ (release 14.0, 2018, Systat-Software Inc.) were utilized. For all tests, a *p* value of 0.05 indicated significance.

Significant changes in cartilage T2* between baseline (t0) and MIs (t1-t3) in the course of the TEFR were calculated by *1-way analysis on variance (ANOVA) with a general linear model for repeated measurements and pairwise post hoc analysis* to calculate the significance between MIs (including the Bonferroni procedure for multiple measurements and a Mauchly test for data sphericity with the Greenhouse-Geisser correction). *Significance of ANOVA inner-subject contrasts* was calculated to detect (linear or polynomial) trends in the T2* values during the progression of the TEFR (accepted for power> 0.8). To determine significant value differences at the last MI (t3) to any maximal change (peak) during exercise (t1-t2) compared with baseline, a *paired (2-tailed) samples t-test with calculation of the effect size according to Cohen*^75^ was performed.

Differences in cartilage T2* between ROIs and zones at the same MI and within the same cartilage segment were calculated by *1-way analysis on variance (ANOVA) without repeated measurements and pairwise post hoc analysis on significance*: the Scheffé test was employed for homogeneous variance (Levene test *p* > 0.1); otherwise, the Bonferroni test was used. To test inter-segmental differences and differences in cartilage T2* between ROIs and zones at the same MI and within the same cartilage segment, a *t-test for independent variables* was performed.

## Results

### Regional cartilage T2* differences

In the right knee, the mean T2* value of the lateral femoral segment was significantly higher than that of the medial segment over the course of the race (t1-t3), while no relevant lateral to medial T2* difference was detected at baseline on either side or at any MI on the left side (see Table 2 and Supplementary Fig. S3). At every MI (t0-t3, tx), the femoral cartilage showed a significantly higher mean T2* signal than the tibial cartilage in both compartments of the left and in the lateral compartment of the right knee (see Table 2 and Supplementary Fig. S4).

**Table 2:** Mean T2* differences [ms] between specific cartilage areas (n_F_ = 17).

	right FTJ					left FTJ				
	t0	t1	t2	t3	tx	t0	t1	t2	t3	tx
**mean differences between lateral and medial knee compartments**^**b**^										
femoral segments	3.1	**5.3**	**6.1**	**6.0**	**2.2**	−1.6	2.3 ^c^	1.9 ^c^	−0.2	1.8
tibial segments	−0.4	1.3	1.9	1.6	0.1	−2.3	−2.0	−1.2	−2.2	0.2
**mean differences between femoral and tibial cartilage segments**^**b**^										
lateral knee compartment	**3.1**	**5.0**	**4.8**	**5.3**	**2.4**	**4.3**	**8.0**	**7.3**	**7.0**	**5.3**
medial knee compartment	−0.4	0.9	0.6	0.9	0.4	**3.5**	**3.7**	**4.2**	**4.9**	**3.3**
**mean differences between superficial and deep cartilage layers within the same cartilage segment**^**b**^										
femoral-lateral	**6.7**	**10.1**	**11.1**	**11.0**	**7.3**	**6.4**	**10.6**^**c**^	**11.0**^**c**^	**10.0**^**c**^	**8.4**
tibial-lateral	**6.8**	**10.8**	**11.8**	**9.8**	**8.5**	**6.1**	**10.1**	**9.9**	**9.7**^**c**^	**6.8**
femoral-medial	**5.5**	**8.8**	**9.1**	**9.3**	**6.9**	**7.6**	**11.2**	**11.9**	**12.4**^**c**^	**8.9**
tibial-medial	**10.2**	**14.8**^**c**^	**14.4**^**c**^	**14.5**^**c**^	**12.6**	**6.3**	**8.9**^**c**^	**8.2**^**c**^	**9.3**^**c**^	**6.2**
**mean differences between ventral, central and dorsal zones within the same cartilage segment**^**a**^										
femoral lateral segment:										
anterior vs. central zone	1.2	0.2	0.1	0.2	−0.1	−0.8	−2.3	−1.0	−1.6	−2.5
anterior vs. posterior zone	−2.3	**−6.7**	−4.6	**−5.5**	−3.1	−2.6	**−5.2**	**−4.3**	−5.0	**−4.8**
central vs. posterior zone	−3.53	**−6.9**	**−4.7**	**−5.7**	−3.0	−1.8	−2.8	−3.4	−3.4	−2.3
tibial lateral segment:										
anterior vs. central zone	0.9	0	1.8	0.5	1.2	0.6	1.9	1.6	1.5	1.1
anterior vs. posterior zone	**−5.8**	**−9.9**	**−8.2**	**−8.2**	**−5.1**	−1.7	−1.7	−3.7	−2.6	**−2.3**
central vs. posterior zone	**−6.67**	**−9.97**	**−10.0**	**−8.8**	**−6.2**	−2.4	−3.6	**−5.4**	**−4.1**	**−3.4**
femoral medial segment:										
anterior vs. central zone	0.1	2.4	3.4	1.6	3.6	3.4	**4.2**	**4.6**	4.1	1.8
anterior vs. posterior zone	**−6.0**	**−5.3**	−3.6	**−6.5**	−1.7	0.9	**−4.5**	−1.6	−1	**−5.0**
central vs. posterior zone	**−6.0**	**−7.7**	**−7.0**	**−8.1**	**−5.3**	−2.4	**−8.7**	**−6.2**	**−5.1**	**−6.8**
tibial medial segment:										
anterior vs. central zone	2.3	2.7	**3.8**	3.6	**4.4**	1.3	1.1	2.8	2.2	1.2
anterior vs. posterior zone	−2.1	**−3.8**	−2.3	−2.3	0.5	0.4	−2.0	−1.1	0.3	−1.1
central vs. posterior zone	**−4.4**	**−6.5**	**−6.1**	**−5.9**	**−3.9**	−0.8	−3.1	**−3.9**	−1.9	−2.2

^a^(one-way) univariate ANOVA, ^b^independent t-test, c: variance homogeneity not given (Levene test <0.05).

Bold fonts show significant differences (*p-value* < 0.05).

At every MI and in all cartilage segments on both sides, the superficial layers had a significantly higher total T2* than the corresponding deep layers (see Table 2 and Supplementary Fig. S3 and S4). Depending on the segment, this mean T2* layer difference was approximately 48% (tibial medial) and 66% (femoral medial) up to 70% (lateral) higher over the course of the race compared with baseline (see Table 2).

In the right knee in all cartilage segments, the mean T2* values showed a significant inter-zone difference at every MI, with the exception of the femoral-lateral segment at baseline (t0). In all segments of the right side, the T2* value was significantly higher in the posterior zone than in the central zone. Tibial lateral at every MI and sometimes in the other segments, it was also significantly higher in the posterior than in the anterior zone (see Table 2). In the left knee, the mean T2* differences between zones within the same cartilage segment were not significant at baseline in any segment and were not significant at t3 in the femoral-lateral segment, at t2 in the tibial-lateral segment or at t1 and t3 in the tibial-medial segment. On both sides, the mean T2* differences between the anterior and central zones were generally not significant.

On both sides, at every MI, a significant difference in mean T2* values between ROIs within the same cartilage segment was detected in all segments (see Supplementary Table S5). As all T2* values of ROIs were significant higher during the race (t1-t3) than at baseline (see Supplementary Table S5), further analyses also showed significantly higher mean T2* differences between zones, layers and segments in the race than at baseline (see Table 2 and Supplementary Fig. S3 and S4).

### Cartilage T2* changes

**Figure 2:**
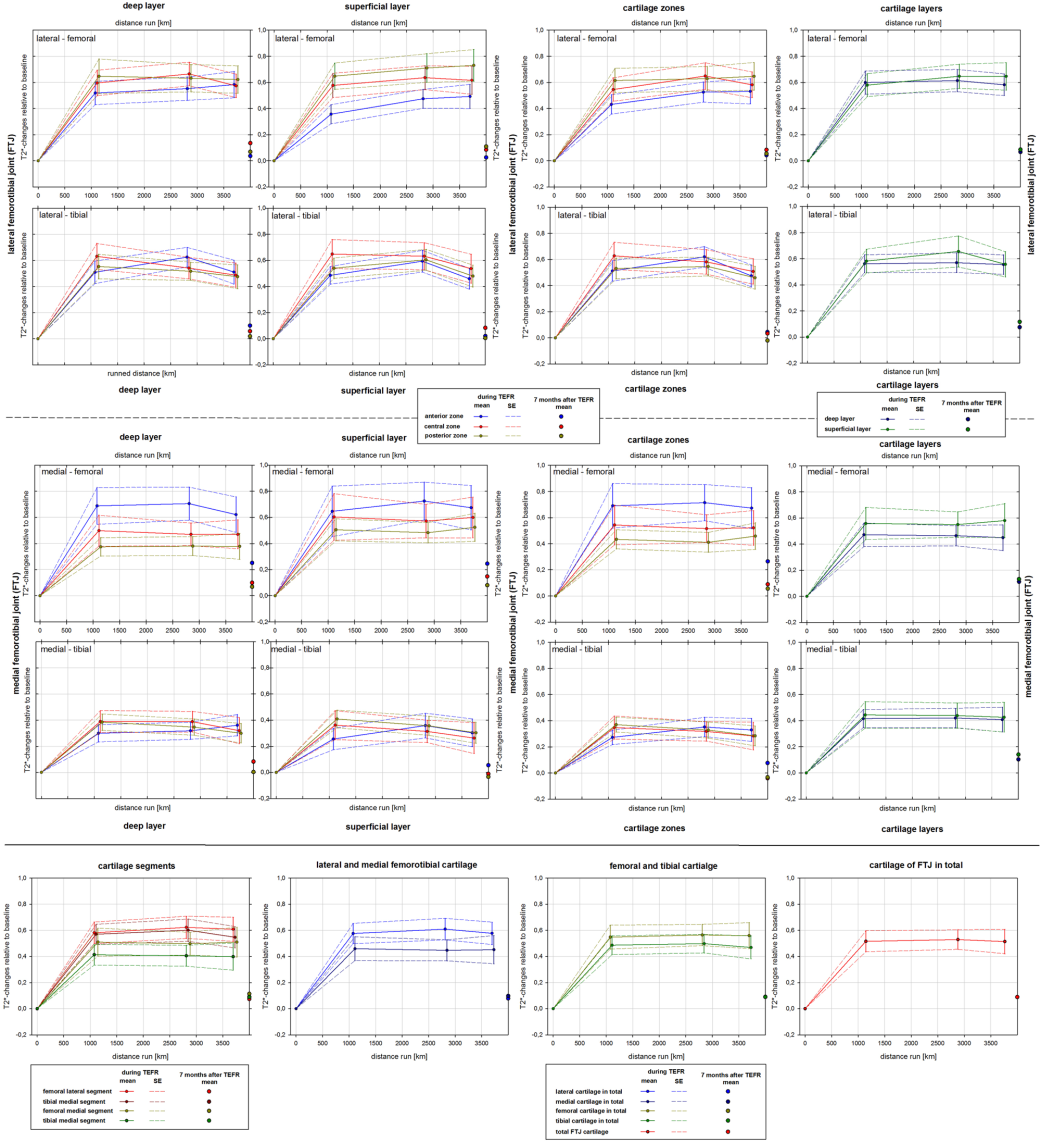
Relative changes of T2* mapping compared to baseline (n_F_ = 17), right knee.

**Figure 3:**
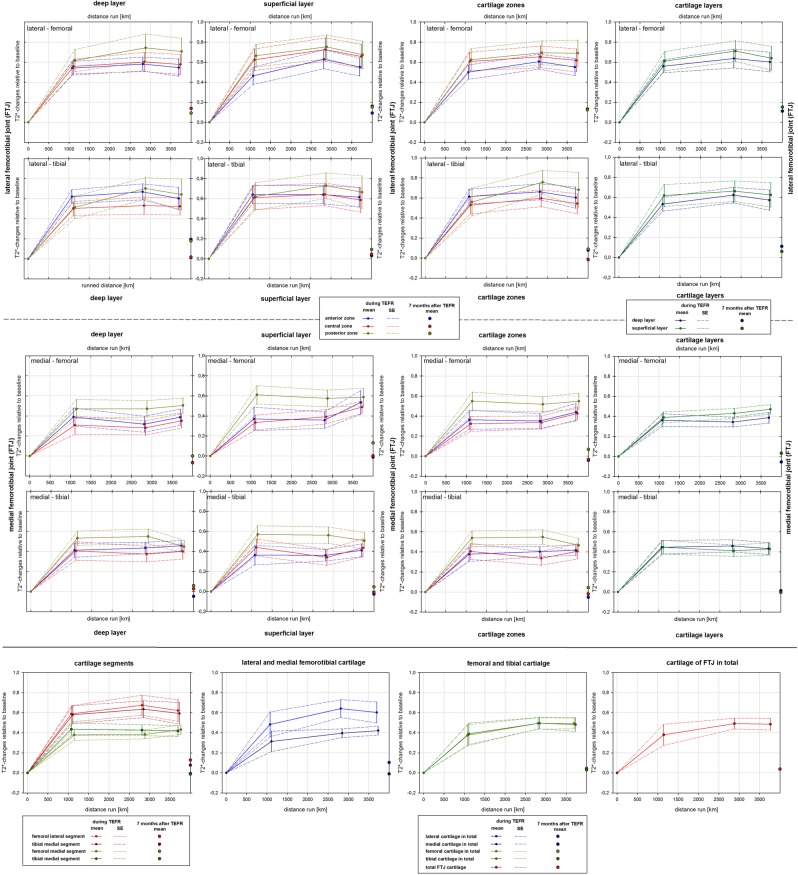
Relative changes of T2* mapping compared to baseline (n_F_ = 17), left knee.

The T2* values changed significantly in all ROIs during the course of the TEFR (see Table 3). The value changes occurred mainly in the first MI with a significant T2* increase: 44.0% femoral-lateral, 42.9% tibial-lateral, 34.9% femoral-medial, and 25.1% tibial-medial (see Figs. 2 and 3 and Supplementary Fig. S3 and S4). Statistical evaluation showed a significant quadratic trend in the T2* curves over the course of the race for nearly all ROIs on both sides (see Table 4). Consecutively, the mean T2* of all cartilage areas showed a significant quadratic trend on both sides (see Figs. 2 and 3 and Supplementary Fig. S3 and S4). With the exception of the anterior and central superficial ROIs of the left medial FTJ, a significant difference in the initial mean T2* increase relative to baseline was not evaluated between any area on both sides (see Figs. 2 and 3). A significant secondary T2* decrease at t3 was detected for the tibial and left femoral segments (mainly in the superficial layer) with a medium to high effect size (see Table 5).

**Table 3:** Changes of intrachondral T2* values [ms] in the course of TEFR (n_F_ = 17).

	segment	ROI	right knee			left knee		
			Mauchly-Test	ANOVA^a^		Mauchly-Test	ANOVA^a^	
			*p-value*	*p-value*	test power	*p-value*	*p-value*	test power
lateral femorotibial joint	femoral lateral	deep-ant.	0.811	**<0.001**	1.000	0.531	**<0.001**	1.000
		deep-central	0.497	**<0.001**	1.000	0.169	**<0.001**	1.000
		deep-post.	0.422	**<0.001**	1.000	0.385	**<0.001**	1.000
		superf.-ant.	0.609	**<0.001**	1.000	0.292	**<0.001**	1.000
		superf.-central	0.686	**<0.001**	1.000	0.083	**<0.001**	1.000
		superf.-post.	0.468	**<0.001**	1.000	0.053	**<0.001**	1.000
	tibial lateral	deep-ant.	0.506	**<0.001**	1.000	0.211	**<0.001**	1.000
		deep-central	**0.030**	**<0.001**^**b**^	1.000	0.484	**<0.001**	1.000
		deep-post.	0.078	**<0.001**	1.000	0.504	**<0.001**	1.000
		superf.-ant.	0.713	**<0.001**	1.000	**0.040**	**<0.001**^**b**^	1.000
		superf.-central	0.370	**<0.001**	1.000	**0.015**	**<0.001**^**b**^	0.999
		superf.-post.	0.467	**<0.001**	1.000	**0.025**	**<0.001**^**b**^	0.995
medial femorotibial joint	femoral medial	deep-ant.	0.881	**<0.001**	1.000	0.393	**<0.001**	1.000
		deep-central	0.493	**<0.001**	0.999	0.201	**<0.001**	0.996
		deep-post.	0.393	**<0.001**	0.999	0.240	**<0.001**	1.000
		superf.-ant.	0.267	**<0.001**	1.000	0.805	**<0.001**	0.999
		superf.-central	**0.049**	**0.001**^**b**^	0.992	0.487	**<0.001**	1.000
		superf.-post.	0.527	**<0.001**	1.000	**0.014**	**<0.001**^**b**^	1.000
	tibial medial	deep-ant.	0.707	**<0.001**	0.994	0.558	**<0.001**	1.000
		deep-central	0.839	**<0.001**	0.992	**0.047**	**<0.001**^**b**^	0.998
		deep-post.	0.753	**<0.001**	1.000	0.651	**<0.001**	0.998
		superf.-ant.	0.778	**0.006**	0.872	0.131	**<0.001**	0.999
		superf.-central	0.499	**0.005**	0.889	0.127	**<0.001**	1.000
		superf.-post.	0.291	**<0.001**	1.000	0.435	**<0.001**	1.000

^a^(one-way) univariate ANOVA for repeated measurements, ^b^with “Greenhouse-Geisser” correction procedure.

Bold fonts show significance (*p-value* < 0.05).

**Table 4:** Analysis on quadratic trends of repeated T2* relaxation time [ms] in the course of TEFR. (univariate ANOVA for repeated measurements, n_F_ = 17).

	ROI	right FTJ		left FTJ	
		medial	lateral	medial	lateral
femoral	deep-ant.	**0.001**	**0.001**	**0.010**	**<0.001**
	deep-central	**0.003**	**0.001**	0.054	**0.001**
	deep-post.	**0.001**	**0.004**	**0.005**	**0.001**
	superf.-ant.	**<0.001**	**0.003**	0.509	**0.004**
	superf.-central	**0.011**	**0.001**	0.067	**0.001**
	superf.-post.	**0.001**	**0.001**	**<0.001**	**0.001**
	deep	**0.001**	**0.001**	**<0.001**	**<0.001**
	superf.	**0.001**	**0.001**	**0.001**	**0.001**
	**total**	**0.002**	**0.001**	**<0.001**	**<0.001**
tibial	deep-ant.	0.024	**<0.001**	**0.003**	**<0.001**
	deep-central	**0.001**	**<0.001**	**0.004**	**0.001**
	deep-post.	**<0.001**	**0.001**	**<0.001**	**<0.001**
	superf.-ant.	0.035	**<0.001**	0.050	**0.001**
	superf.-central	**0.006**	**0.001**	**0.006**	**0.001**
	superf.-post.	**0.000**	**<0.001**	**<0.001**	**0.001**
	deep	**<0.001**	**0.001**	**<0.001**	**0.001**
	superf.	**0.006**	**0.002**	**0.001**	**0.001**
	**total**	**0.001**	**0.001**	**<0.001**	**0.003**
Femoral segments		**<0.001**		**0.001**	
Tibial segments		**<0.001**		**<0.001**	
Total		**0.002**	**<0.001**	**0.001**	**0.001**
Total FTJ		**<0.001**		**0.001**	

Bold fonts show significant p-values with high test power (>0.8).

**Table 5:** Analysis (paired *t*-test) on secondary T2* decrease [ms] at last MI (t3). (n_F_ = 17).

		ROI	right knee		left knee	
			*p-value*	*d*	*p-value*	*d*
lateral femorotibial joint	femoral lateral segment	deep-ant.	0.042	0.45	0.009	0.47
		deep-central	**0.028**	0.71	0.006	0.47
		deep-post.	**0.027**	0.63	0.009	0.43
		superf.-ant.	0.060	0.38	0.015	0.44
		superf.-central	0.030	0.45	0.010	0.48
		superf.-post.	0.032	0.44	0.010	0.47
		deep	**0.050**	0.64	0.012	0.44
		superf.	0.037	0.45	0.011	0.48
		**total**	**0.042**	0.54	**0.008**	0.55
	tibial lateral segment	deep-ant.	**0.006**	0.62	**0.009**	0.55
		deep-central	**0.022**	0.63	0.010	0.49
		deep-post.	**0.014**	0.72	0.006	0.44
		superf.-ant.	**0.009**	0.73	0.039	0.47
		superf.-central	**0.012**	0.64	0.015	0.42
		superf.-post.	**0.006**	0.78	0.005	0.40
		deep	**0.034**	0.66	0.016	0.44
		superf.	**0.036**	0.62	0.016	0.32
		**total**	**0.33**	0.63	0.010	0.42
medial femorotibial joint	femoral medial segment	deep-ant.	**0.003**	0.67	0.004	0.27
		deep-central	0.016	0.49	0.022	0.35
		deep-post.	0.009	0.46	0.002	0.46
		superf.-ant.	0.008	0.42	0.027	0.29
		superf.-central	0.012	0.38	0.023	0.25
		superf.-post.	0.029	0.41	0.010	0.41
		deep	**0.012**	0.50	**0.007**	0.50
		superf.	0.020	0.38	**0.028**	0.69
		**total**	0.016	0.41	0.014	0.45
	tibial medial segment	deep-ant.	0.013	0.45	0.043	0.44
		deep-central	**0.003**	0.65	0.024	0.35
		deep-post.	**0.003**	0.70	**<0.001**	0.89
		superf.-ant.	**0.001**	0.57	**0.036**	0.59
		superf.-central	**0.001**	0.67	**0.029**	0.53
		superf.-post.	**0.004**	0.62	**0.010**	0.66
		deep	0.010	0.49	0.013	0.49
		superf.	**0.014**	0.52	0.012	0.39
		**total**	0.011	0.45	**0.020**	0.63
Femoral segments			0.035	0.46	**0.027**	0.51
Tibial segments			**0.025**	0.52	**0.029**	0.55
Lateral FTJ			**0.043**	0.59	0.017	0.47
Medial FTJ			0.014	0.43	0.040	0.45
Total FTJ			0.033	0.48	**0.041**	0.50

Bold fonts show significant *p-value*s with middle (d > 0.5) to high (d > 0.8) effect size (Cohen’s d).

### T2* reaction of nearby structural osteochondral lesions

**Figure 4:**
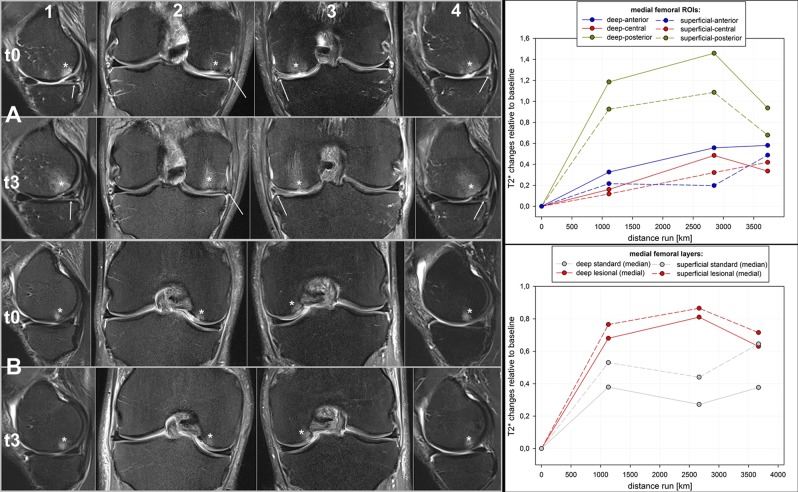
Osteochondral lesions (*) in the FTJ at baseline (t0) and last MI (t3) with no relevant signal changes throughout TEFR (sagittal TIRM: (1) right side, (4) left side; coronal PDfs: (2) right side, (3) left side). (**A**) 58-year-old male finisher (t3: stage 57/3,971 km run); bilateral medial meniscal lesions in the cornu posterior, grade 3 on the right and grade 2 on the left side (*arrows*) with adjacent femoral chondral lesions Outerbridge grade 3 on the right, and Outerbridge grade 4 on the left side with focal femoral subchondral bone edema on both sides (*): mean T2* increases more in lesional posterior ROIs, but shows secondary decrease at t3. Non lesional anterior and central ROIs show only slight continuous mean T2* increase. (**B**) 48-year-old male non-finisher (t3: stage 63 / 3,669 km run); bilateral osteochondral lesions at baseline (Outerbridge grade 2–3) with a focal subchondral bone edema (*) located femoral medial: mean T2* increases more in lesional medial layers but shows secondary decrease at t3. Non lesional median layers show only slight continuous mean T2* increase.

In 2 male subjects with femoral medial focal grade 3–4 OA at baseline, with no further structural changes throughout the TEFR, the corresponding regional cartilage showed significantly higher absolute T2* values at baseline and a comparatively higher initial mean T2* increase (at t1) and higher secondary mean T2* decrease (at t3) compared with all other non-lesion ROIs (see Fig. 4).

## Discussion

We found a significant T2* increase in all cartilage areas of the FTJ in the first third of the TEFR. After a “steady state” in the middle of the race and at approximately 3,500 km of the run, the T2* signal showed a detectable decreasing trend in many, mostly tibial cartilage regions (see Table 5, Figs. 2 and 3). To the best of our knowledge, this is the first and only prospective MRI study that analysed the cartilage response of a large number of human knee joints during an ultra-run load of almost 4,500 km; therefore, a comparative discussion is only possible considering previous studies of animal models with similar distances or studies of shorter running distances in humans.

### Immediate, primary T2^(^*^)^ decrease with rapid recovery

In static and acute loading studies^38,76^ and in most post-race studies of short- (5 km) to long-distance runs (marathons) that employed MRI within 1 hour^30–33,35–37,77^, a pressure-dependent significant T2 decrease was observed in all cartilage layers. With cyclical loading, cartilage deformation produces an increase in superficial collagen anisotropy and a concomitant decrease in (free) cartilage water, leading to lower post-race T2 values. For example, Mosher *et al*.^36^ found a relevant initial T2 decrease in superficial cartilage (tibial: 1–3 ms, femoral: 2–4 ms) and no T2 change in the deep layer in subjects after a 30-min run. This immediate T2 response recovers rapidly after 30 min up to a few hours^78–81^. However, we could not detect such primary T2* decreases at any MI (t1-t3), since our first data were acquired at t1 after a distance run of 962–1,325 km (stage 14–20), at which time the effects of long-distance running dominate (masked) the immediate T2-response.

### Long-distance, primary T2(*) increase

Animal studies have shown that a more strenuous long-distance running burden leads to GAG decrease in the main weightbearing cartilage areas^82–84^. Only 2 studies have focused on T2 mapping from 1 hour up to less than 24 hours after a single marathon race in humans^34,58^. Both reported significantly increased T2 values in knee cartilage, in contrast to all published studies with the first follow-up performed after less than 1 hour. Using high-field MRI, Luke *et al*.^58^ found not only significantly higher T2 values but also equivalent higher intra-chondral T1rho values in the FTJ 48 hours after a marathon, postulating that this signal elevation is mainly caused by a proportional GAG decrease^45^. Animal long-term running studies (young dogs: 1 year, 40 km/day) showed that if the interstitial environment of chondrocytes is disturbed, high fluid pressure leads to disorganization of the collagen and proteoglycan network and structural changes in chondral fibrillation^83,85^. Therefore, the initial significant T2* increase in the cartilage areas of the FTJ after the 1,100 km TEFR run may not only be explained by the loss of spatial collagen structure; rather, it may also be caused by increased free cartilage water due to GAG depletion. Since no relevant morphometric and structural changes were observed during the T2* increase in the first third of the TEFR^86^, our data may be explained by changes in chondral permeability and respective ion flow, which reached a new equilibrium from stage to stage.

During the TEFR a significant mean T2* difference between ROIs was detected in all segments (see Table 2). Since all these regional T2* differences were higher throughout the race than at baseline (see Supplementary Table S5), this finding applies to all zones, layers and segments (see Table 2 and Figs. 2 and 3). However, we could not determine significant difference in T2* increase between cartilage areas (see Figs. 2 and 3).

There is a typical spatial variation in T2 values in healthy cartilage. For example, lower T2 values are found in the deeper radial layer, where the collagen fibres are highly ordered, and elevated T2 values are found in the superficial layer with less collagen organization^41,87,88^. Mosher *et al*.^36^ observed that this spatial dependency of cartilage T2 is a function of the distance from bone, even in recreational runners. This finding is explained by cartilage biochemistry: quantitative microspectroscopy has shown a significant running-induced decrease in GAG content in the weight-bearing superficial layer of the lateral femoral (5–13%) and tibial cartilage (5–35%) of the canine FTJ^55,85,89^. The deformation rate of cartilage increases due to disorganization or lesion of the superficial collagen network due to reduced GAG content^55^, which contributes to instant cartilage stiffness through osmotic pressure^90^. Since cartilage stiffness is directly related to GAG content^91^, the cartilage equilibrium shear modulus also decreases significantly in the lateral FTJ (12–14%)^85^. Consistent with the fact that cartilage areas with regularly high loads are stiffer than areas with lower loads^92^, we found significant mean T2* differences between the superficial and deep layers throughout the TEFR in all cartilage areas of the FTJ (see Table 2 and Supplementary Fig. S3 and S4).

Zonal dependency of cartilage T2 has also been observed in several studies, and we found higher T2* values in the posterior zone than in the central and anterior zones, being significant in the right FTJ. As Mosher *et al*.^36^ observed in normal running, we also found significantly higher T2 values and greater T2* variation in femoral cartilage than in tibial cartilage at all MIs during the MSUM. In humans, Subburaj *et al*.^42^ indicated a greater load on the medial compartment of the FTJ during running and, therefore, that the medial cartilage is stiffer and shows a higher isotropy of collagen matrix. With increasing load, the contact area and pressure seem to increase, especially in the lateral compartment of the FTJ^93^. This might explain why the left medial segments showed a significantly lower average T2* increase than the lateral segments in our study group (see Table 2 and Figs. 2 and 3).

### Ultra-long distance, secondary T2^(^*^)^-decrease (“recovery”)

In the FTJ cartilage, the long-distance T2 increase within 48 hours after a single marathon recovered to baseline after 3 months^58^. As our subjects continued to run daily ultra-marathons up to 64 stages (more than 2 months) without sufficient recreation time, T2* recovery could not be expected until t3. At the final MI (t3), we measured a significant secondary T2* decrease in the tibial and left femoral segments (mainly in the superficial layer) with a medium to high effect size (see Table 5). Our results on ankle and hindfoot cartilage T2* behaviour at TEFR^70,71^ showed the same reaction in the axial loaded joints: a primary T2* increase as the first long-distance T2* response after 1,100–2,400 km run^85^ but an earlier and stronger secondary T2* decrease (“recovery”) as an ultra-long distance T2* response after more than approximately 2,000–2,500 km run.

A reasonable explanation is reorganization of the articular cartilage collagen network^94^ and reduction in free water because it gets the ability to go back in chemical bond due to recovery of GAG content^95^. A positive effect of joint loading (moderate to ultra-long running) on chondrocyte function with increased GAG and collagen synthesis has been reported in multiple animal studies^94,96–100^, and increased GAG has been reported in a few studies on running^95^ and weight-bearing^101^ in human knee cartilage. Since increasing hydrostatic pressure up-regulates proteoglycans and type-II collagen mRNA expression^102^, de novo syntheses of proteoglycans will be initiated, and their increased concentration has been shown to impede hydraulic fluid flow^103^. This mechanism shields the collagen-proteoglycan matrix from high stress and protects against cartilage degeneration and OA^94^. In addition to these MR findings, changes in the serum concentrations of cartilage biomarkers of TEFR subjects also showed that articular cartilage is able to adapt during an MSUM^104^. The observation of a secondary T2* decrease at t3 in cartilage regions with preexisting higher-grade cartilage degeneration after the above-average signal increase in 2 of our subjects (see Fig. 4) may also support the hypothesis of ongoing T2* recovery in the FTJ of ultra-runners with an ongoing running burden beyond a distance of 3,500 km.

As animal experiments have shown that ongoing ultra-long load bearing exercises minimize the development of OA in rats^96^ and dogs^83^, and as our collective of highly adapted and experienced ultra-runners^105^ did not show increased cartilage degeneration, we found significant recovery of T2 values after TEFR (tx), as reported in all three published post-marathon studies with a second follow-up^34,39,40^. Thus, ongoing ultra-endurance running burden does not correspond to increased OA or overuse lesions in the human knee joint when relevant cartilage injuries or malalignments are absent^16,106^. Therefore, despite the problems that have been discussed with overuse injuries in ultra-marathon running^107–114^, MSUMs do not seem to have a negative effect, but rather a favourable influence, on cartilage.

Due to the small number of non-finishers (3) and subjects with dominance of the left leg (3), sufficient data for statistical comparison of finishers or dominance of the right leg were not available (Table 1). Arokoski *et al*.^55^ showed side dependence in their experimental animal studies and tried to explain this by a side-dependent adaptation of the knee cartilage to mechanical loadings. The different degrees and types of loading on articular surface areas in the canine FTJ correspond to our observation of right to left side differences of the T2* cartilage response to the ultra-long-distance TEFR: a significant inter-zone T2* difference in all segments was found on the right but not regularly on the left, and a significant T2* increase in the medial compared with the lateral segments was found on the right but not on the left. As we also found comparable chondral T2* side differences in the ankle and hindfoot joints of TEFR subjects^70,71^, which may be due to the right leg experiencing higher load and ground repulsion forces during the stance phase. The asymmetry of locomotion, which is based on the tendency of humans to use one leg preferentially during motor movements^115–117^, could possibly also be present during MSUM running. However, this explanatory model remains speculative, especially since this study has some limitations that do not allow any further reliable statistical evaluations about relevant causes and influencing factors with regard to the detected T2* reactions during the TEFR.

### Limitations

This study is subject to possible selection bias, as participants had been running ultra-marathons for almost a decade prior to enrolment. Therefore, these results may not be generalizable to novice individuals who begin ultra-long-distance running. The age dependency of the articular cartilage matrix content^118^ is also a source of possible bias on the T2* measurement in this study, but we do not consider its relevance to be very strong, since the age range among the subject groups was not large (Table 1). In addition, the test subjects did not adhere to a strict resting phase before the baseline measurement but carried out light running training (only a few kilometres per day during the last week), which could result in a discrete bias: slightly higher T2* values measured at baseline compared with a collective with a sufficiently long resting phase before the start of the TEFR. It must be assumed that the described immediate T2 decrease is present in parallel with the mechanism of the primary long-distance T2 increase. The amount of this “masking” effect and the attenuation of the real T2* increase and the influence of the stage run burden on T2* mapping in general is difficult to estimate^79^. Including a control group was not feasible due to race conditions and the study setting.

The results of this mobile MRI-based field study allow us to refute a relevant negative influence of ultra-long running distances on knee joint cartilage in ultra-marathoners^106^. However, in addition to the factor of running distance, there are other external running-specific characteristics (e.g., competition runners, mountain running with inclines and descents, cross-country running with enhanced multiaxial stress on the knee joints., interval running with partially high running speed, etc.) that may increase the risk of developing knee OA^119,120^. These factors were not included in our investigation and require further clarification in other studies^121^.

## Conclusions

The measured chondral T2* values showed similar physiological regional distribution patterns and relative differences between cartilage areas of the FTJ as in normal, healthy non-endurance and other athletic collectives, although these values were higher throughout the TEFR than at baseline. Due to initial chondral matrix degradation within the first 1,100 km of running, a significant, primarily non-regional-related T2* increase occurred in all cartilage areas of the FTJ, caused by collagen disorientation and decrease and GAG depletion. As a further increase in the second half of the TEFR was not observed, we do not expect further softening or degradation of the FTJ cartilage with ongoing running burden over ultra-lengthy periods extending to 4,500 km. However, we expect the FTJ cartilage matrix to be able to reorganize itself, as the T2 * curve showed a decreasing trend (recovery) after 3,500 km. Therefore, our results are not consistent with the hypothesis that ultra-long-distance running in specifically trained athletes may be associated with increased incidence and severity of knee joint OA. The observed side differences in regional T2* changes are in line with the hypothesis of the ‘braking’ limb and also indicate side dominance of one leg in MUSM runners, which must be verified by specific studies in the future. This study shows the feasibility of mobile functional MRI for precise quantitative non-invasive measurements to evaluate the biochemical long-term effects of MSUM running on knee joint cartilage.

## Supplementary information

Supplementary information

## Data Availability

Additional materials, data and associated protocols are promptly available to readers without undue qualifications in material transfer agreements.
