# Supplementary material for: A mobile MRI field study of the biochemical cartilage reaction of the knee joint during a 4,486 km transcontinental multistage ultra-marathon using T2* mapping
Source: Sci Rep. 2020 May 18;10:8157. doi: 10.1038/s41598-020-64994-2 (PMC7235258; doi:10.1038/s41598-020-64994-2)
Supplement: Supplementary file 1 — Supplementary information [file 41598_2020_64994_MOESM1_ESM.pdf]

A mobile MRI field study of the biochemical cartilage reaction of the knee joint during a 4,486 km transcontinental multistage ultra-marathon using T2* mapping

Uwe Schütz, Martin Ehrhardt, Sabine Göd, Christian Billich, Meinrad Beer, Siegfried Trattnig

Table S1: Interrater reliability (Pearson CC) of T2* measurements (n=152)

cartilage segment	femoral		tibial	
	lateral	medial	lateral	medial
**Cartilage ROIs:**				
**deep-ant.**	0.892*	0.944*	0.945*	0.943*
**deep-central**	0.931*	0.934*	0.966*	0.903*
**deep-post.**	0.967*	0.937*	0.953*	0.889*
**superf.-ant.**	0.941*	0.933*	0.940*	0.847*
**superf.-central**	0.967*	0.940*	0.961*	0.862*
**super.-post.**	0.981*	0.789*	0.954*	0.789*
**Cartilage layers:**				
**deep**	0.955*	0.956*	0.884*	0.875*
**superficial**	0.975*	0.947*	0.810*	0.564*
**Cartilage zones:**				
**anterior**	0.846*	0.927*	0.930*	0.900*
**central**	0.900*	0.948*	0.915*	0.919*
**posterior**	0.956*	0.890*	0.835*	0.869*
Total segments	*0.977**	*0.964**	*0.883**	*0.869**

* Correlation (Pearson CC) is high (r > 0.8) and significant on a niveau of p<0.01 (two-sided test).

Table S2: Mean cartilage area sizes [mm²] drawn for T2* evaluation in the course of the race (SD).

cartilage segment	total segment		anterior zone		central zone		posterior zone	
	right	left	right	left	right	left	right	left
**femoral lateral**	152.4 (9.4)	148.3 (12.2)	36.4 (4.5)	33.3 (3.6)	55.5 (5.4)	56.9 (4.7)	60.6 (4.2)	60.3 (4.7)
**tibial lateral**	160.1 (11.2)	167.8 (14.2)	44.2 (4.6)	43.5 (4.8)	69.2 (7.7)	74.8 (8.8)	46.6 (4.5)	49.5 (7.3)
**femoral medial**	180.4 (12.5)	183.3 (10.2)	38.5 (5.0)	42.8 (5.2)	72.1 (10.8)	70.0 (9.5)	69.8 (5.6)	70.6 (5.8)
**tibial medial**	165.3 (10.1)	165.7 (9.8)	35.7 (4.4)	39.0 (5.6)	71.1 (9.2)	67.9 (7.5)	58.5 (5.1)	58.8 (5.5)
total:	***658.3 (29.6)***	***656.4 (40.8)***	***154.8 (12.1)***	***158.6 (12.4)***	***267.9 (22.8)***	***269.5 (19.5)***	***235.6 (12.3)***	***239.2 (16.1)***

Figure S3: Absolute values of T2* mapping (n_F=17). right side

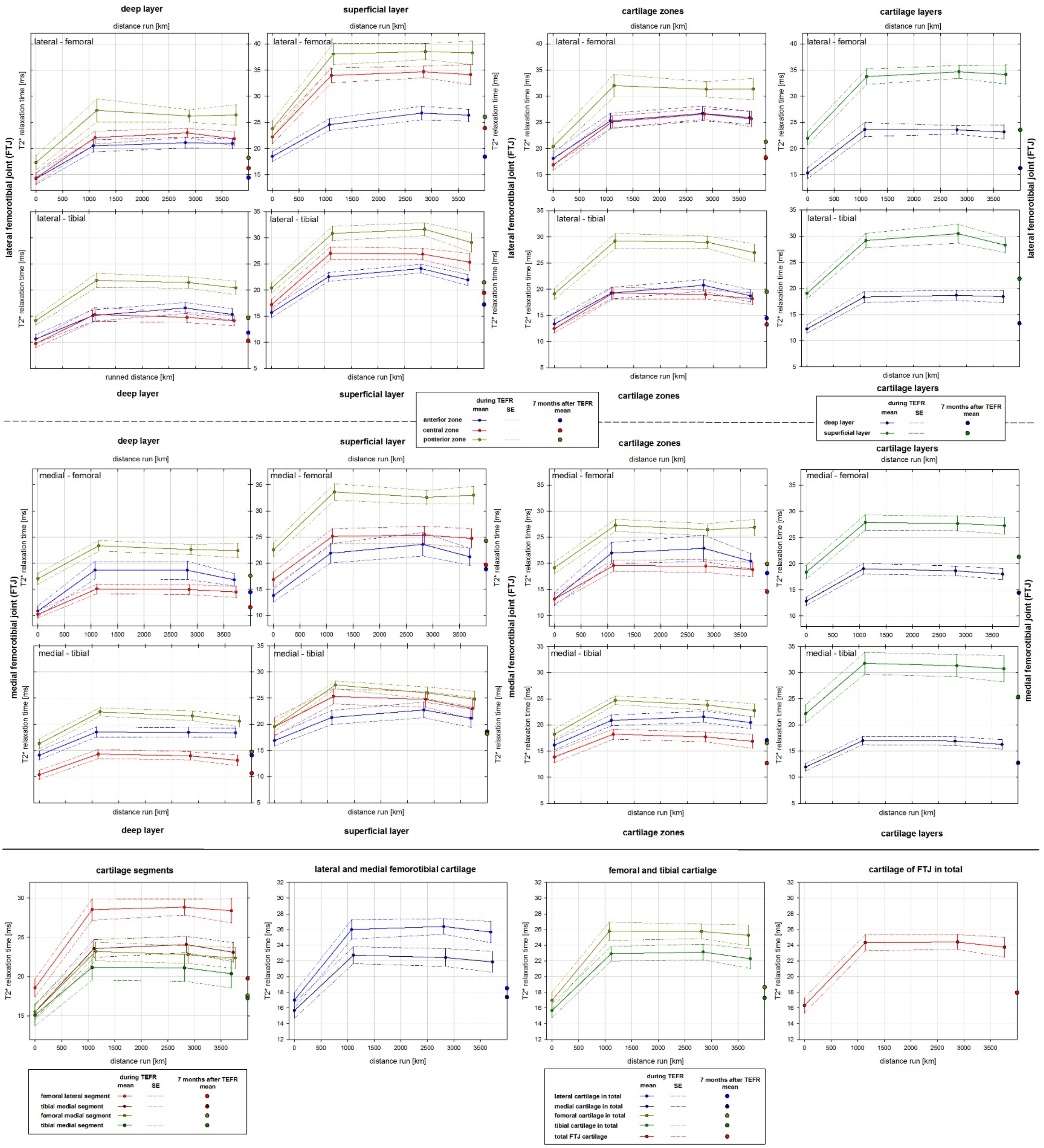

Figure S4: Absolute values of T2* mapping (n_F=17). left side

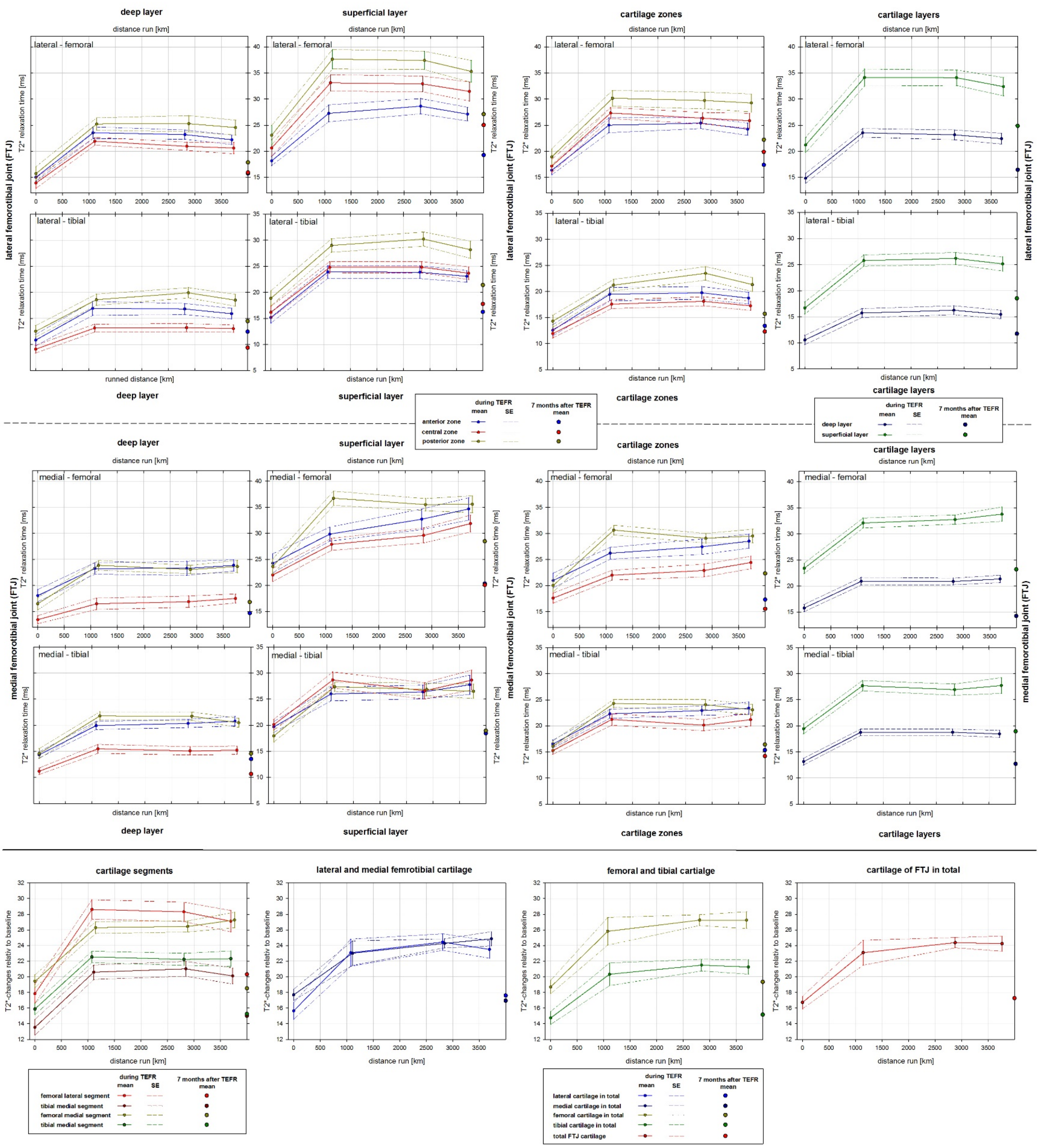

Table S5: Mean T2* differences [ms] between cartilage ROIs within the same cartilage segment (n_F=17, univariate ANOVA).

ROI	right FTJ					left FTJ				
	**t0**	**t1**	**t2**	**t3**	**tx**	**t0**	**t1**	**t2**	**t3**	**tx**
**femoral lateral segment**										
superf.- vs. deep-ant.	4.2	4.1	5.7	5.4	4.0	3.1	3.7	5.4	4.9	3.5
superf.-ant. vs. deep-central	4.1	2.5	3.8	4.5	2.2	4.2	5.4	**7.7**	6.5	3.3
superf.-ant. vs. deep-post.	1.2	-2.7	0.6	-0.1	0.2	2.4	2.0	3.4	2.6	1.4
superf.-central vs. deep-ant.	**8.0**	**13.5**	**13.5**	**13.2**	**9.5**	5.6	**9.6**	**9.7**	**9.3**	**9.3**
superf.-central vs. deep-central	**7.8**	**11.9**	**11.7**	**12.3**	**7.7**	**6.7**	**11.2**	**12**	**10.9**	**9.1**
superf.-central vs. deep-post.	4.9	6.7	**8.4**	7.7	**5.7**	4.9	**7.9**	**7.6**	6.9	**7.2**
superf.-post. vs. deep-ant.	**9.5**	**17.6**	**17.4**	**17.3**	**11.6**	**8.1**	**14.1**	**14.2**	**13.1**	**11.4**
superf.-post. vs. deep-central	**9.4**	**16.0**	**15.5**	**16.4**	**9.8**	**9.2**	**15.7**	**16.5**	**14.7**	**11.2**
superf.-post. vs. deep-post.	**6.5**	**10.8**	**12.3**	**11.9**	**7.8**	**7.4**	**12.4**	**12.1**	**10.8**	**9.2**
superf.-central vs. superf.-ant.	3.7	**9.4**	**7.9**	7.8	**5.5**	2.5	5.8	4.3	4.3	**5.8**
superf.-post. vs. superf.-ant.	5.3	**13.5**	**11.7**	**11.9**	**7.6**	4.9	**10.4**	**8.8**	**8.2**	**7.8**
superf.-post. vs. superf.-central	1.6	4.1	3.9	4.2	2.2	2.4	4.5	4.5	3.8	2.1
deep-central vs. deep-ant.	0.1	1.6	1.9	0.9	1.8	-1.1	-1.6	-2.2	-1.6	0.2
deep-post. vs. deep-ant.	3.1	6.8	5.1	5.4	3.8	0.7	1.7	2.1	2.3	2.1
deep-post. vs. deep-central	2.9	5.2	3.2	4.5	2.0	1.8	3.3	4.3	3.9	1.9
**tibial lateral segment**										
superf.-ant. vs. deep-ant.	**5.1**	**7.4**	**7.5**	**6.6**	**5.4**	4.3	**7.0**	**7.0**	**7.2**	**3.8**
superf.-ant. vs. deep-central	**5.9**	**7.2**	**9.3**	**7.8**	**6.9**	**6.1**	**10.8**	**10.6**	**10.0**	**6.9**
superf.-ant. vs. deep-post.	1.5	0.7	2.7	1.5	2.6	2.6	5.3	4.0	4.6	1.8
superf.-central vs. deep-ant.	**6.6**	**11.9**	**10.3**	**10.0**	**7.6**	5.3	**7.9**	**8.0**	**7.8**	**5.3**
superf.-central vs. deep-central	**7.4**	**11.7**	**12.1**	**11.2**	**9.1**	**7.1**	**11.6**	**11.6**	**10.6**	**8.4**
superf.-central vs. deep-post.	3.0	5.2	**5.4**	4.9	**4.8**	3.7	**6.2**	4.9	5.2	3.3
superf.-post. vs. deep-ant.	**9.7**	**15.7**	**15.1**	**13.7**	**9.6**	**8**	**12.1**	**13.4**	**12.3**	**8.9**
superf.-post. vs. deep-central	**10.6**	**15.5**	**16.8**	**14.9**	**11.1**	**9.7**	**15.8**	**17**	**15.1**	**12.0**
superf.-post. vs. deep-post.	**6.2**	**9.0**	**10.2**	**8.7**	**6.8**	**6.3**	**10.4**	**10.3**	**9.7**	**6.9**
superf.-central vs. superf.-ant.	1.5	4.5	2.8	3.4	2.2	1.0	0.9	1.0	0.6	1.5
superf.-post. vs. superf.-ant.	**4.7**	**8.3**	**7.5**	**7.1**	**4.2**	3.7	5.1	**6.4**	5.1	**5.1**
superf.-post. vs. superf.-central	3.2	3.8	4.8	3.7	2.0	2.7	4.2	**5.4**	4.5	**3.6**
deep-central vs. deep-ant.	-0.9	0.2	-1.8	-1.2	-1.5	-1.7	-3.8	-3.6	-2.9	-3.1
deep-post. vs. deep-ant.	3.5	**6.7**	4.9	5.1	2.8	1.7	1.7	3.1	2.6	2.0
deep-post. vs. deep-central	**4.4**	**6.5**	**6.7**	6.3	**4.3**	3.4	5.4	**6.7**	5.5	**5.1**
**femoral medial segment**										
superf.-ant. vs. deep-ant.	3	3.2	5	4.4	4.4	6.2	**6.6**	**9.4**	**10.8**	5.7
superf.-ant. vs. deep-central	3.6	6.8	**8.7**	6.7	**7.3**	**10.8**	**13.4**	**15.8**	**17.2**	**8.9**
superf.-ant. vs. deep-post.	-3.2	-1.4	1.0	-1.2	1.3	**7.8**	**6.0**	**9.7**	**11.0**	3.5
superf.-central vs. deep-ant.	**6.0**	6.5	6.7	**7.9**	5.2	4.0	4.6	6.3	**8.0**	5.4
superf.-central vs. deep-central	**6.7**	**10**	**10.4**	**10.3**	**8.1**	**8.6**	**11.4**	**12.7**	**14.4**	8.6
superf.-central vs. deep-post.	-0.1	1.8	2.8	2.3	2.1	5.5	4.0	**6.5**	**8.2**	3.3
superf.-post. vs. deep-ant.	**11.7**	**14.9**	**14.0**	**16.2**	**9.8**	5.6	**14.5**	**12.2**	**11.7**	**13.8**
superf.-post. vs. deep-central	**12.4**	**18.5**	**17.7**	**18.5**	**12.7**	**10.2**	**21.2**	**18.6**	**18.1**	**17.0**
superf.-post. vs. deep-post.	**5.5**	**10.3**	**10.0**	**10.6**	**6.7**	**7.1**	**13.9**	**12.5**	**11.9**	**11.7**
superf.-central vs. superf.-ant.	3.1	3.2	1.8	3.5	0.8	-2.3	-2.0	-3.1	-2.8	-0.3

superf.-post. vs. superf.-ant.	**8.8**	**11.7**	**9.0**	**11.8**	5.4	-0.6	**7.9**	2.8	0.9	8.1
superf.-post. vs. superf.-central	**5.7**	**8.5**	7.2	**8.3**	4.6	1.6	**9.8**	5.9	3.7	8.4
deep-central vs. deep-ant.	-0.6	-3.6	-3.7	-2.3	-2.9	-4.6	**-6.8**	**-6.4**	-6.3	-3.2
deep-post. vs. deep-ant.	**6.2**	4.7	4.0	5.6	3.1	-1.6	0.6	-0.2	-0.2	2.1
deep-post. vs. deep-central	**6.8**	**8.2**	**7.7**	**7.9**	**6.0**	3.0	**7.4**	6.2	6.1	5.4
**tibial medial segment**										
superf.-ant. vs. deep-ant.	2.8	2.8	4.3	2.8	4.5	**5.4**	**6.1**	**6.0**	**7.0**	**4.9**
superf.-ant. vs. deep-central	**6.5**	**7.0**	**8.8**	**8.0**	**8.0**	**8.5**	**10.5**	**11.3**	**12.6**	**7.8**
superf.-ant. vs. deep-post.	0.6	-1.1	1.2	0.6	3.9	**5.1**	4.2	4.7	**7.3**	3.9
superf.-central vs. deep-ant.	**5.4**	**6.8**	**6.3**	4.7	4.3	**5.8**	**8.7**	**6.3**	**7.9**	**5.4**
superf.-central vs. deep-central	**9.2**	**11.0**	**10.8**	**9.9**	**7.7**	**8.9**	**13.2**	**11.5**	**13.4**	**8.2**
superf.-central vs. deep-post.	3.3	3.0	3.2	2.4	3.6	**5.6**	**6.9**	4.9	**8.2**	**4.3**
superf.-post. vs. deep-ant.	**5.5**	**9.0**	**7.5**	6.4	4.1	3.6	**7.4**	**6.5**	5.7	**5.4**
superf.-post. vs. deep-central	**9.2**	**13.2**	**12**	**11.7**	**7.5**	**6.8**	**11.9**	**11.8**	**11.3**	**8.3**
superf.-post. vs. deep-post.	3.3	**5.2**	4.4	4.2	3.4	3.4	**5.6**	5.1	6.0	**4.4**
superf.-central vs. superf.-ant.	2.6	4.0	2.0	1.9	-0.3	0.4	2.7	0.2	0.9	0.5
superf.-post. vs. superf.-ant.	2.7	**6.2**	3.2	3.6	-0.4	-1.7	1.4	0.5	-1.3	0.5
superf.-post. vs. superf.-central	0.0	2.2	1.2	1.8	-0.2	-2.2	-1.3	0.3	-2.2	0.1
deep-central vs. deep-ant.	-3.7	-4.2	-4.5	-5.2	-3.4	-3.1	-4.5	-5.3	-5.6	-2.9
deep-post. vs. deep-ant.	2.2	3.8	3.1	2.2	0.6	0.2	1.8	1.4	-0.3	1.0
deep-post. vs. deep-central	**5.9**	**8.1**	**7.6**	**7.5**	4.1	3.4	**6.3**	**6.6**	5.3	3.9

* **Bold fonts** show significant differences (*p-value*<0.05)
